# *‘This is the core of what we do’*: a qualitative study of social prescribers’ attitudes towards spiritual health training and their future training needs

**DOI:** 10.1186/s12875-025-02979-8

**Published:** 2025-11-06

**Authors:** Mark Adley, Alexandra Thompson, Philip Mordue, Amy O’Donnell, Barbara Hanratty, Ishbel Orla Whitehead

**Affiliations:** Population Health Sciences Institute, Faculty of Medical Sciences, Campus for Ageing and Vitality, Newcastle-upon-Tyne, NE4 5PL UK

**Keywords:** Spiritual health, Social prescribing, Primary care, Training, Development, Peer learning

## Abstract

**Background:**

Despite the evidence supporting the importance of spiritual health to people’s wellbeing across diverse fields of treatment, the topic of spiritual health is not currently mentioned in National Health Service (NHS) training materials for social prescribers. Previous research with social prescribers has identified a need for training around spiritual health in primary care. This study sought in-depth understanding of these training needs and how they may be met.

**Method:**

Semi-structured interview data specific to the subject of training needs were extracted from interview data from a wider study, which explored barriers and facilitators to spiritual health discussions within social prescribing. UK-based social prescribers aged 18 + working in primary care were recruited purposively from different geographic areas, with data collected between December and February of 2025. An inductive, iterative approach was taken to the thematic analysis of data.

**Results:**

Findings were generated from interviews with 12 participants with three main themes: *the value and need for spiritual health training*, *approaches to training*, and *the value of learning from peers and patients*. However, while social prescribers interviewed broadly recognised the benefits that spiritual health training could bring to their work with patients, there were some who did not feel this was relevant to their role.

**Conclusions:**

Participants identified how integrating spiritual health discussions into social prescribing improved not only patients’ health but also their own knowledge and skills. In-person training was widely felt to be appropriate for discussions around spiritual health. However, participants noted the limitations of one-off training sessions within this context, and highlighted the potential benefits of ongoing learning within the workplace. Peer learning appears to be a valuable and useful method of training for the topic of spiritual health, recognising social prescribers’ understanding of holistic health and focusing on the relevance of spiritual health to patients. Including the topic within NHS competency frameworks and training materials would also greatly support the relevance of spiritual health to social prescribing roles.

**Supplementary Information:**

The online version contains supplementary material available at 10.1186/s12875-025-02979-8.

**Table:** 

What is ‘spiritual health’? Spiritual health is a broad concept, as diverse as people themselves. The authors use a definition of spiritual health developed by UK General Practitioners and further developed with social prescribers: self-actualisation, peace, purpose and meaning; transcendence, connectivity and relationships beyond the self; and expressions of spirituality.	

## Background

Social prescribing is a major policy focus within the National Health Service (NHS) in the UK, as part of a wider shift towards addressing health inequalities via the provision of Universal Personalised Care [1]. There is emerging literature in the UK, with reviews exploring the impact of social prescribing initiatives upon general patient groups [2], or specific cohorts such as people experiencing loneliness [3], mental ill health [4], long term conditions [5], or autistic adults [6]. While social prescribing initiatives have demonstrated improvements across a range of outcomes and patient groups, many of the reviews highlight that small study sizes and the lack of standardised definitions and metrics hamper the ability to draw conclusions about their impact.

One of the core concepts within the training and guidance for social prescribers is to find out from patients *what matters to you?* [1], with this question framed in contrast to the biomedical model in which patients are defined by their illness (or what’s the matter *with* you?). This approach is aligned with the concept of salutogenesis – the promotion of health and well-being [7, 8] – which includes building upon personal resources and inner strengths in order to thrive across physical, cognitive, social, and spiritual dimensions of health [9, 10].

Spiritual health is important to people’s mental health and overall wellbeing [11–13]. Unmet spiritual health needs are associated with reduced patient wellbeing [14, 15]. Ignoring or failing to address spiritual health needs can therefore come at a cost. This cost is not only to individual patients’ wellbeing [16], but also to communities via increased levels of social isolation [17], and the public purse, for example resulting from lost working hours, and increased healthcare use and mortality and morbidity rates [18–20].

**Fig. 1:**
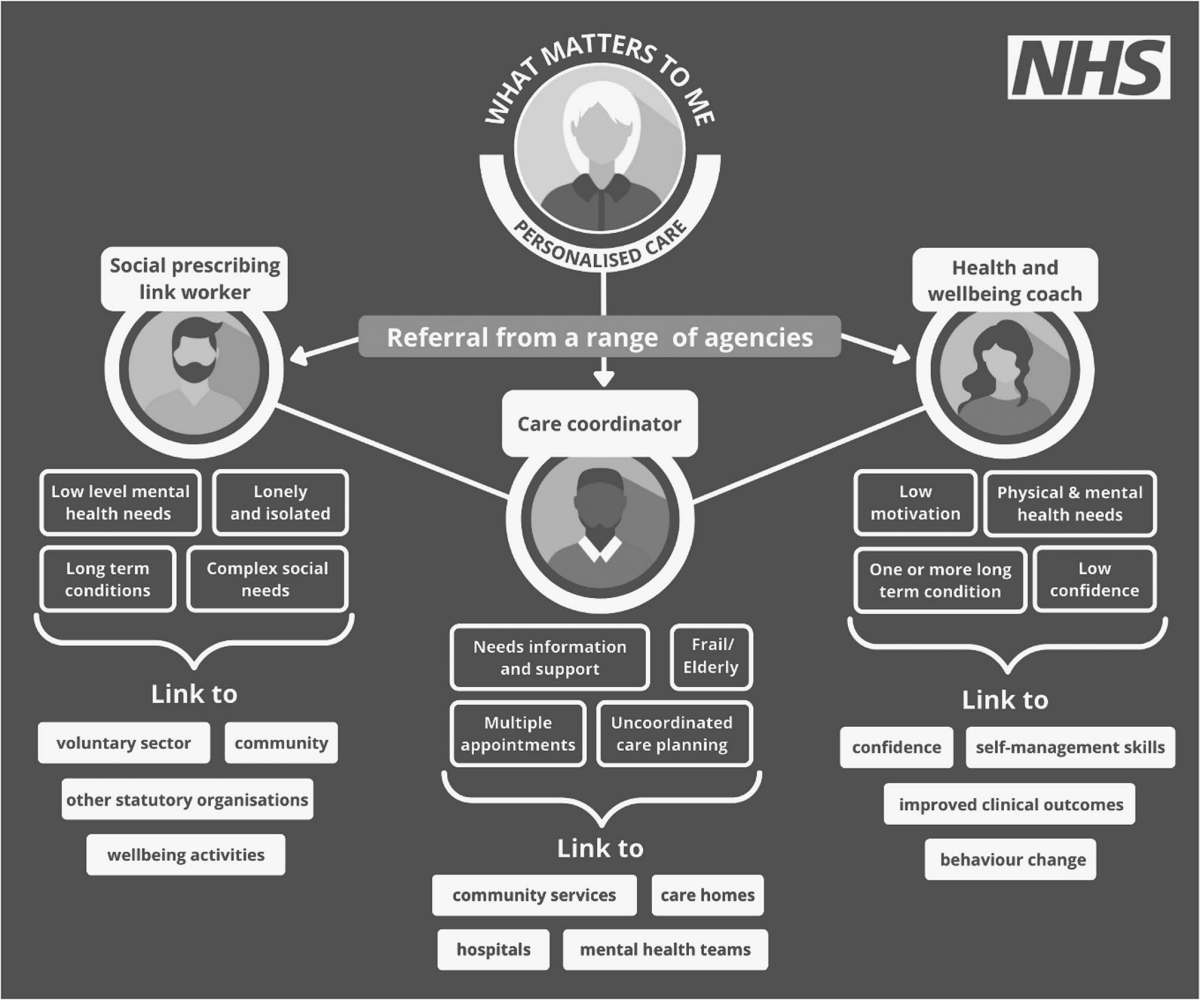
The different types of support provided by social prescribing link workers, care coordinators, and health and wellbeing coaches [21]

Although this paper uses the term social prescribers, in the UK this is a blanket term that includes a number of roles including social prescribing link workers, care coordinators, and health and wellbeing coaches (Fig. 1). Across these roles, people may also have management responsibilities.

There is currently no legally-required qualification for any of the three personalised care roles shown in Fig. 1, with social prescribers coming from varied backgrounds and not required to have formal training in healthcare [22]. The NHS workforce development framework provides core competencies and standards for social prescribing practice, including staff support, supervision, and learning and development, workforce development, and support for improved quality and consistency and reduced variation in outcome [21, 23, 24]. However, despite their focus on social prescribers’ communication and relationship-building skills and the ability to deliver personalised care, none of these documents mention spiritual health. This topic is also absent from the NHS e-learning training programme that social prescribing link workers are required to complete [21].

Learning and development for the three personalised care roles varies widely across the nations of the UK, with training provided by both NHS and third sector organisations. NHS England, in partnership with the NHS and NHS Health Education England, provides training in multiple formats. The FutureNHS website hosts training and development resources for social prescribers, including webinar recordings and a discussion forum [25]. Within the third sector, charities such as the Social Prescribing Network [26] and National Academy for Social Prescribing [27] provide resources for social prescribers, including webinars and e-learning. There are also locally-decided assessment tools and outcome measures, many of which use various images depicting outcomes ‘stars’ or ‘wheels of wellbeing’, with spiritual health included in some, but not all of these (see Table 1).

**Table 1:** Examples of 'spokes' in the visual models of wellbeing used in the UK

Examples of ‘spokes’ of the ‘wheels of wellbeing’ used in social prescribing in the UK. These spokes support a holistic approach to care by identifying areas of strength and need, guiding tailored non-clinical interventions. 1. Physical Wellbeing – This includes physical health, diet, exercise, sleep, and management of long-term conditions [1–6]. 2. Emotional/Mental Wellbeing – Covers mental health, stress, mood, and resilience [3–6]. 3. Social Wellbeing – Focuses on relationships with family, friends, and community engagement [1–3, 5, 6]. 4. Spiritual Wellbeing – Includes aspects of meaning and purpose, religious or cultural identity, and opportunities for fulfilment such as volunteering or purposeful activity [2–4]. 5. Work and Learning – Refers to employment, education, and skill development [1, 2, 4–6]. 6. Financial Wellbeing – Relates to income, benefits, budgeting, and debt management [1, 2, 5, 6]. 7. Environmental Wellbeing – Includes housing quality, neighbourhood safety, and access to nature or green spaces [1, 3, 5, 6]. 8. Creativity and Leisure – Encompasses participation in hobbies, creative pursuits, and leisure time for enjoyment and relaxation [1, 5, 6]. 9. Self-Care and Independence – Refers to managing daily tasks, autonomy, and accessing support when needed [6].	

Examples [6] Outcomes Star. Well-being Star 2025. Available from: https://outcomesstar.org/well-being-star/

The need for training around the topic of spiritual health within primary care settings has been identified by General Practitioners (GPs) and social prescribers. Only half of the 177 GPs surveyed in England felt comfortable discussing spiritual health with patients [28]. A lack of training was identified as a source of this discomfort, with the study recommending dedicated training on this topic. Recently, social prescribers also highlighted this same need for spiritual health training; noting that one of the barriers to spiritual health discussions was a lack of confidence that the topic was part of their role (unpublished data, Whitehead IO, et al., May 2025).

Notably, there is no mention of spiritual health within development frameworks for the three social prescribing personalised care roles. This omission may suggest that, despite the evidence highlighting the importance of spiritual health to patient wellbeing, this significant element of patient care is not being endorsed within social prescribing interventions.

## Aims and objectives

This study therefore aimed to explore the spiritual health training needs of social prescribers working within primary care in England and Wales. The objectives were:

- To explore social prescribers’ past experiences of training around spiritual health and their training needs.
- Identify issues that might attract or discourage them from attending training.
- Explore potential content and format for a future training offer.

## Methods

### Design and setting

A qualitative study design was selected to explore participants’ training needs. Semi-structured interviews were used to capture some of the context and cultures within which study participants worked, and to allow for research questions to evolve during the study [29]. Data specific to the subject of training needs were extracted from interview data from a wider qualitative study, led by IOW, which had explored barriers and facilitators to spiritual health discussions within social prescribing.

The current study sought to explore social prescribers’ training needs around discussion of spiritual health with patients. Interviews used open-ended questions (see Supplemen­tary Material for topic guide) that were broadly grouped as follows:

*If training was going to be developed around spiritual health within social prescribing*,* what do you think should and shouldn’t be included?*

*What would be helpful for you to have in that training: What kind of information or resources would be helpful?*

*What format should such training take?*

*What are your views on that kind of training - do you think there’s a need for it?*

### Recruitment and sampling

Participants aged above 18 years were recruited purposively from different ethnic groups and geographic areas to provide diverse views and experience. Recruitment took place via a previously circulated survey, shared via NHS email to GP practices, NHS bodies and research networks, via Twitter/X, and professional networks and newsletters. Participants in the survey were asked to contact the team if they were willing to be interviewed, and interviewees were selected from this list to provide a varied group in terms of geography and gender. Participants were given a £20 gift voucher in thanks. Only UK-based social prescribers working in primary care with direct patient contact were eligible to take part. All participants provided written informed consent to participate in the study and for the publication of its findings.

### Data collection techniques

Data were collected via semi-structured interviews conducted between December and February of 2025 using Microsoft Teams [30] or Zoom [31] videoconferencing software. Audio recordings were saved onto a secure server before being transcribed, either by a professional transcription service or using software-based transcription, with identifiable data anonymised or removed after transcription.

### Public and patient involvement (PPI)

PPI work had taken place prior to the wider interview study with members of VOICE, a network of public, patients, and carers. The subject of spiritual health elicited mixed views from public contributors. They expressed the importance of person-centred care, and that spiritual health has its place within whole person care, but also expressed concerns regarding extra burden on over-stretched primary care staff. Integrating this feedback into the wider study contributed to its development, and to IOW’s concept of social prescribers being best placed to have spiritual health discussions with patients within primary care.

### Ethics

Ethical approval was obtained from Newcastle University on 21^st^ August 2024 and HRA approval was obtained on 25^th^ October 2024, IRAS number 347636. Ethical standards were observed in accordance with the Declaration of Helsinki.

### Data analysis

**Fig. 2:**
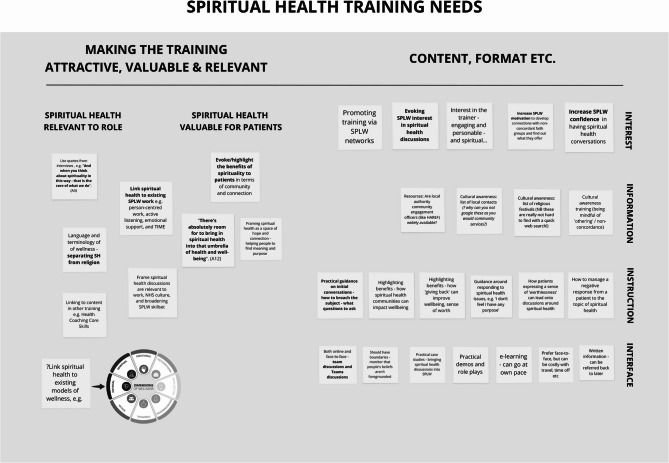
The researchers’ digital whiteboard created in Miro software formed the initial coding framework

The process of coding took place in a heuristic, iterative manner, aligned with Braun and Clarke’s six phases of thematic analysis [32]. Familiarisation with the data began with IOW and AT conducting participant interviews. Additional familiarisation took place between 6th January and 3rd February 2025 during which IOW, MA, and AT analysed between three and four interviews each. The researchers then met on 3rd February to share and discuss their findings. From these discussions initial codes were generated, with Miro software [33] used to create a digital whiteboard onto which virtual ‘post-it’ notes could be added (Fig. 2). Themes were then reviewed for relevance, leading to refinement of the coding system such as the merging of the themes *Information* and *Instruction* (see Fig. 2). Interview data were then re-analysed in MAXQDA software [34] according to these themes. Ongoing analysis and discussion resulted in the generation of the three themes presented below.

## Results

Twelve participants took part in interviews, with details of participant demographics in Table 2. Three main themes were generated: *the value and need for spiritual health training*,* approaches to training*, and *the value of learning from peers and patients*. Themes supported the relevance of the topic of spiritual health to participants’ social prescribing role, and the potential value of related training. Discussions with colleagues were described as helpful ways of sharing information and receiving support.

**Table 2:** Participant demographics (NP = no preference/not present/not provided)

ID	Age	Sex	Nationality	Ethnicity	Region	Role title (self-defined)	Approx years in role	Setting
Participant 1	59	F	British	White British	Oxfordshire	Social prescriber	3.5	Semi-rural
Participant 2	24	F	British	White British	West Midlands	Enhanced social prescriber/Wellbeing Coach	1	NP
Participant 3	NP	NP	NP	NP	NP	Social prescriber	1.5	NP
Participant 4	47	F	British	Black British/Afro-Caribbean	London	Social prescriber - team lead	4.5	Urban
Participant 5	51	F	British	White British	Highlands	Social prescriber	3	Rural
Participant 6	NP	NP	NP	NP	NP	Social prescriber	3	Suburban
Participant 7	55	F	British	White British	Merseyside	Manager of specialist social prescribing service	4	Semi-rural
Participant 8	28	F	Australian	White Other	Surrey	Social prescriber	2	Suburban
Participant 9	44	F	British	White British	North East England	Social prescriber/care coordinator	1.5	Rural
Participant 10	57	F	British	White Mixed	West Yorkshire	Social prescriber	8	Urban
Participant 11	60	M	British	White British	South West Scotland	Community link worker	2	Rural
Participant 12	NP	NP	NP	NP	NP	Social prescriber	6	Urban

### The value and need for spiritual health training

Not all the social prescribers interviewed expressed an interest in training around spiritual health. When asked whether such training would be useful Participant 1 replied simply: *‘No*,* I don’t think so*,* no… because spiritual- it’s somebody’s choice.’* Others viewed these discussions as outside of their remit as social prescribers, skirting around the topic of spiritual health which was at times conflated with religion: ‘*But like I say*,* because a lot of the people*,* their automatic thought is*,* it’s religion. And I don’t want to be speaking about that*’ (Participant 6).

Finding out ‘what matters’ to patients is central to the social prescribing role, and participants noted that training could provide a platform for spiritual health discussions. For Participant 10, spiritual health training offered the opportunity to address issues around disconnection and isolation, central to the work of social prescribers:

*‘This [spiritual health training] is something that’s going to be out there and available to people*,* because it needs to be*,* there’s definitely a gap there…I think this is just so needed*,* any form of reconnection with self*,* with others*,* is definitely needed’.*

Social prescribers interviewed highlighted the importance of spiritual heath to patients, with Participant 4 also suggesting that is the responsibility of workers to bring up the topic: *‘I think it’s really important that we have those type of conversations… because the person probably may want to say something but isn’t sure how you’re going to respond, or maybe this isn’t the space to have this type of conversation so they don’t tell you anything but*,* actually*,* it could be the very core of how you help them move on… it’s very remiss of us if we don’t try and have a conversation’.*

Participants also noted personal benefits from spiritual health discussions, leading to self-reflection and *‘…understanding your own*,* your own faith*,* you know*,* and*,* looking at your own faith in a different*,* from a different angle… As your awareness grows*,* spirituality*,* understanding of spirituality changes’* (Participant 12).

Many participants felt that spiritual health was a valuable part of their work. For Participant 3 and others, supporting patients with their sense of meaning, purpose, and connection was embedded within the social prescribing role:

*‘But I think it makes a lot of sense… in terms of*,* you know… doing something with meaning and purpose. And when you think about spirituality in this way. That is the core of what we do’.*

### Approaches to training

Many of the participants asked for information about faith and religious groups to be included within any spiritual health training offer, for example *‘it might be useful to know a bit more about different faith groups that are available and what they do and how to connect to them’* (Participant 1). While social prescribers might generally look for information about local organisations, Participant 9 felt that standard approaches to finding community services might not be applicable:

*‘It would be quite nice to know what’s available out there for people because sometimes it’s quite difficult with religious things to*,* to look up online because it’s quite difficult. You know*,* they don’t always have websites and things like that’.*

Practical guidance on how to have constructive conversations around spiritual health was also felt to be of benefit. Participant 5, for example, said that *‘it would be really nice to actually have prompt questions. You know*,* open-ended questions that*,* that would just open up that conversation or get that person and maybe sort of*,* sort of some guided yeah*,* questions for discussion with people’.* A more rigid script to guide spiritual health conversations was also requested:

*‘I think if we’re going to have training on something*,* a practical “This is how you could conduct a conversation. This is like your opening line and this is how you…” like I think that would be*,* that would open up so much more’ (Participant 2).*

Some social prescribers expressed concerns that discussions around religion might lead to difficult conversations with patients, and wanted advice around this: *‘Perhaps some people may have had negative experiences*,* so it might be quite triggering*,* so perhaps how to manage those conversations as well’* (Participant 8). Other social prescribers interviewed felt more confident within spiritual health discussions and wanted to broaden their skillset: *‘What kinds of questions can we ask? What kinds of*,* yeah*,* how can we guide that a bit further so we can go a bit deeper into those conversations maybe?’* (Participant 4).

While there was no consensus about the preferred format of the training, in-person training was widely felt to be appropriate for discussions around spiritual health:

*‘When you’re doing face to face [training] that is*,* because obviously spirituality*,* a lot of it*,* it’s all about being together in a group… then that’s probably a bit a better way to do it because people are going to be there and they’re going to be with each other*,* which you know*,* spirituality is very community’* (Participant 9).

Others noted the limitations of one-off training and highlighted the potential benefits of ongoing learning within the workplace: *‘So, whatever would sort of work, whether it’s an online training and meet, have a group of people and you’re sort of meeting them regularly, once every few weeks or a month’.* (Participant 8).

### The value of learning from peers and patients

Becoming more confident when discussing spiritual health with patients helped to develop social prescribers’ skills: *‘so you tend to develop of art of having those conversations and*,* again*,* that’s really about what matters to the person’* (Participant 4). Participant 2 stated that opening discussions around spiritual health *‘better informs my practice’* while Participant 10’s understanding of spiritual health led to the topic being integrated into their local assessment tool:

*‘Spiritual wellbeing is one of the aspects that’s on there… I wrote some little descriptors and for spiritual wellbeing I basically put in a nutshell it’s about understanding beliefs*,* values, and ethics that help to shape the way you live and guide you in life… so just talking about how*,* you know*,* the things that we do… the procedures if you like of working with patients in my organisation is that we use this wellbeing tool’.*

Social prescribers interviewed highlighted the value of learning not only from each other, but also from patients. For Participant 12, discussions with patients had led to a reassessment of their own personal beliefs:

'Just learning from what they’ve got to say. It’s interesting… and, looking at your own faith in a different, from a different angle, if you know what I mean… As your awareness grows, spirituality, your understanding of spirituality changes, you know?'

Peer support allowed social prescribers to discuss challenges, share experiences, and seek advice from colleagues:

*‘I guess having sort of sessions where you’re learning a little bit more about how to have those conversations and then perhaps ideas of how to put it into practice and then almost having that peer support chat of*,* “okay*,* how’s it been*,* have you had any cases where you did use that? How did it go?” So sort of*,* yeah*,* sort of putting it into action and discussing how it goes*,* as well.’* (Participant 8).

The benefits of simply discussing the topic of spiritual health with colleagues were mentioned, for example that it would be: ‘*nice to have the kind of space to actually talk about it [spiritual health] and think about it and explore it a bit’* (Participant 5). Participant 2 felt that conversations around the topic of spiritual health with colleagues could increase their confidence with patients: *‘us having conversations so that we’re prepped to have conversations with the client’*. Given the potentially emotive nature of the subject, a facilitated learning environment was suggested by several participants:

*‘This subject is so*,* it’s one of them. You know*,* people can go off on one or*,* you know*,* people can also just retreat into themselves and listen and just think*,* actually*,* "I don’t want to talk about what my thoughts are on this because it’s all, you know"’.* (Participant 9).

Peer-led approaches also provided opportunities to learn from each other’s perspectives and experiences, in comparison to other formats:

*‘Because you can try out different ways of*,* of addressing things*,* and also*,* everybody else can listen*,* can’t they*,* and then they can learn from*,* from seeing… and being able to learn from other people. I mean*,* if you’re doing an e-learning*,* you’re not learning from anybody else*,* are you?’* (Participant 7).

## Discussion

Religious and spiritual health factors play an important role within health, with positive associations found across physical, mental, and social health domains [35, 36]. However, the omission of spiritual health training for health professionals, including social prescribers, leaves staff *‘ill-prepared to address the religious or spiritual component of their patients’ life experiences’* [37]. The current study therefore sought to explore the spiritual health training needs of social prescribers, to identify issues that might attract or discourage them from attending, and to consider the format of future training.

Social prescribers in the current study were broadly enthusiastic about the prospect of receiving spiritual health training. The topic of spiritual health appeared to hold more value in specific settings such as in end of life care, and the two social prescribers who worked in specialist cancer services expressed more comfort with the topic of spiritual health training. As the other participants worked more generally it was not possible to draw conclusions regarding the influence of workplace roles and settings. However, not all participants expressed interest, or felt that the topic of spiritual health was relevant to their role. This is a new finding, but it has similarities to previous survey responses from GPs [38].The participants in the current study may have felt challenged about their identities as social prescribers: some participants defended the person-centred nature of their roles, stating that raising the topic of spiritual health with patients would counter a person-led approach. Other participants may have felt that the suggestion that training was needed in spiritual health implied they were not doing their jobs well. Thus the idea that they might benefit from training could evoke, as described within Hermans’ work on the dialogical self, *‘a reciprocal stance*,* a counter-position’* [39].

The current study’s theme of the value of peer learning may offer an approach to engaging those who were disinterested in attending spiritual health training. It has been recommended elsewhere that: *‘For those who might be reluctant to attend seminars on spiritual issues*,* being open to learning from colleagues offers a viable educational option’* [40]. Peer learning involves both cognitive processes, in which people make meaning within their own minds, and social processes, in which knowledge is constructed within social and cultural interactions and practices [41, 42]. Learning takes place in cooperative situations with peers in similar social groupings, without positions of authority [43]. Peer learning could challenge some of the stigma and potential pluralistic ignorance (the belief that while *I* feel it’s important, nobody else does) around the topic of spiritual health. When participants discuss learning about spiritual health from patients, they are describing reflexive experiential learning [44, 45] in line with Knowles’ Andragogical principles [46] that learning needs to be experiential, relevant, and problem-centred.

Our study findings highlight the need for more prominent information about the role spiritual health plays in health and wellbeing, and the economic costs of not addressing patients’ spiritual health needs. The findings also suggest that within healthcare settings, colleagues who have identified the relevance, importance, and value of spiritual health within their role might be well-placed to facilitate supportive discussions around this topic. Social prescribers come from a wide range of professional backgrounds, and the responsibility for having spiritual health discussions with patients should not sit with them alone. Awareness of individual limitations and team strengths is a vital component of effective health and social care, and nurses, doctors, or other professionals with strong spiritual or cultural competence may be best placed to lead such discussions.

### Future directions

Many of the social prescribers in this study identified the importance of spiritual health to their role, which may mean that this was not a representative sample. It will be important to ensure that any future training is attractive to those who do not see the topic as relevant to their role, that it brings the value and need of addressing patients’ spiritual health to the foreground, and makes clear how the topic of spiritual health is aligned with social prescribing roles. Social prescribers work alongside their patients, evoking *what matters to* patients rather than telling them [21]. Decision-making and plans are made together, rather than being dictated by a clinician in a position of responsibility. It follows that training for social prescribers might benefit from a congruent approach, with training delivered on a voluntary basis using materials that highlight the potential for skills development [47].

Within narrative pedagogy, learning and teaching are viewed as co-occurring, collaborative, and communal experiences; its approach considers the impact of teaching *as* learning, and reframes learning as *listening* [48]. Narrative pedagogy highlights the importance of stories that have personal significance, and meaning that is shared through lived experiences [49]. There were powerful narratives within the current study’s interviews, with participants articulating the relevance, importance, and value of spiritual health within social prescribing. Narratives such as these, shared by peers, might be embedded into the design and delivery of spiritual health training. This is aligned with the recent work by Mazzoli and colleagues [50] who presented a narrative-based learning framework that can be implemented within person-centred healthcare.

Many primary care practices have ‘time out’ sessions in which the practice closes, and time can be allocated to training and development. These might provide the ideal setting for peer learning sessions around spiritual health. However, difference of power or status can create a significant barrier to peer learning [51]. This has implications for the success of this approach, given the hierarchical nature of primary care settings [52]. Future studies might evaluate peer learning opportunities within primary care. For example, whether peer learning within primary care is better suited to smaller groups of people who share the same or similar roles, rather than mixed groups where levels of power and influence may vary.

### Strengths and limitations

Most of the social prescribers interviewed within this study spoke positively about the prospect of attending spiritual health training. However, this may reflect the recruitment of people with an interest in the topic of spiritual health. Although this recruitment approach generated meaningful data on the topic, the findings might not be typical of the body of social prescribers. Similarly, the lack of religious and ethnic diversity amongst the interviewees should be addressed in future studies. The study would have benefitted from the collection of more participant data prior to interviews: not only around cultural characteristics but also around the scope of participants’ varied roles (e.g. social prescriber, health and wellbeing coach, care coordinator), and a summary of their previous roles and disciplines. This might have offered the potential for deeper analysis, set against previous role experience. There was however a richness to the qualitative data, and the iterative nature of its analysis led to identification of the theme of the value of peer learning. This study has also brought to light a significant omission within social prescribers’ current training and competency frameworks, and the potential benefits that spiritual health training might bring both to patients and social prescribers themselves.

### Implications for research and practice

Previous research has suggested strategies to improve integration of spiritual health discussions within primary care. Training around spiritual health improved practitioners’ confidence in discussing the topic with patients, and practitioners who reflected on their own beliefs were found to be best equipped to engage in spiritual health discussions [53, 54]. In line with Knowles’ andragogical theory, training should be relevant, self-directed, and experience-based, with learning opportunities that acknowledge participants’ existing values and encourage real-world application [46]. Simply initiating conversations about spiritual health, both with patients and colleagues, emerges as a strong predictor of future engagement; highlighting the importance of reducing stigma through normalising dialogue. Reflective spaces which allow healthcare practitioners to process the sensitive nature of spirituality in a workplace setting [55] exem­plify experiential learning, and may support professional growth.

While some social prescribers interviewed perceived religion to be outside the scope of primary care, peer learning and informal discussion can serve as inclusive, non-threatening routes to explore the topic. Emphasising personal growth and development aligns this approach with adult learning theory [56] and fostering a patient-centred approach to spiritual health. However, this raises the issue of stigma towards discussions around spiritual health within healthcare. Aligned with the literature [57], social prescribers in the current study felt that the topic of religion was not to be discussed within their role in primary healthcare. Researchers and practitioners might reflect not only on the treatment model in which social prescribers work, but also on the underlying assumptions about religion and spirituality that are implied. For those social prescribers interviewed who were less interested in formal spiritual health training, peer learning based around narrative pedagogy and reflective problem-based approaches learning could offer a promising way to bring this topic into primary care settings. Participants highlighted how spiritual health is already integral to their role: supporting people around isolation, developing social connections, and identifying purpose and meaning within ‘what matters to’ them. However, the NHS could take a significant step towards the removal of stigma around the topic of spiritual health by including the importance of spiritual healthcare within the workforce development frameworks, guidance documents, and training for social prescribers – and other primary care staff.

## Conclusions

Although there is growing recognition that spiritual health plays a key role in overall wellbeing, it has yet to be integrated into training for all care providers in primary care. Whilst a majority of social prescribers interviewed were supportive of spiritual health training this was not a unanimous view. Peer learning is a potential way to bridge these gaps. The stories shared by social prescribers in this study suggest that spiritual health training – and the conversations it sparks – might improve patient wellbeing. Regular ‘time out’ sessions already held in many primary care practices could provide an opportunity for this kind of peer learning. Future research could explore how peer-learning discussions about spiritual health can fit into primary care, and whether these conversations should happen within groups who hold similar roles, for example social prescribers and health and wellbeing coaches, or across primary care teams as a whole.

## Supplementary information

Supplementary Material 1.

## Data Availability

Data are saved on Newcastle University secure servers and may be available in negotiation with this paper’s last author. While participants were not asked to consent to allowing public sharing of this data, data may be available upon reasonable request to orla.whitehead@newcastle.ac.uk.

## Abbreviations

GP: General practitioner
NHS: National Health Service

## Declarations

Abbreviations.

GP (General Practitioner).

NHS (National Health Service).

Consent for participation and publication.

All participants provided written informed consent to participate in the study and for the publication of its findings.
